# Predatory publishing and global scholarly communications

**DOI:** 10.29173/jchla29907

**Published:** 2025-12-01

**Authors:** Heather Cunningham

**Affiliations:** Gerstein Science Information Centre University of Toronto Toronto, ON, Canada

Berger M. **Predatory publishing and global scholarly communications**. Chicago, IL: Association of College and Research Libraries, a division of the American Library Association; 2024. ACRL Publications in Librarianship No. 81. Softcover: 350 p. ISBN: 978-0-8389-8955-5. Price: USD$115.00. Available from: https://alastore.ala.org/predatory-publishing-and-global-scholarly-communications.

Monica Berger’s *Predatory publishing and global scholarly communications* offers a thorough exploration of the complex issue of predatory publishing within the larger scholarly communication landscape. Berger, an Instruction and Scholarly Communications Librarian and professor at the New York City College of Technology, City University of New York, has published extensively over the past 12 years on topics including predatory publishing, open access, and scholarly communication. Far from presenting a simple dichotomy of “good” versus “bad” publishers, Berger frames predatory publishing as a “wicked problem” which captures its complexity and resistance to straightforward solutions. The book situates this phenomenon within a broader scholarly ecosystem shaped by competing motivations among authors, editors, and publishers, as well as systemic pressures from academia’s reward structures, which often prioritize quantity over quality. Predatory publishing can be reframed as fulfilling a need. Berger also examines the roles of profit-driven publishing models, open access, and the commodification of academic work, reminding readers that predatory publishing predates Beall’s List and includes earlier forms such as vanity and advocacy publishers.

Importantly, Berger highlights that even large, mainstream publishers may engage in questionable or exploitative practices, such as double-dipping by charging both authors (via article processing charges (APCs) for open access) and libraries (via subscription fees), while also employing surveillance practices that track user behaviour and data through academic platforms to monetize usage analytics. She draws on a range of scholarly voices who note the difficulty in defining what constitutes a predatory journal. Nevertheless, Berger, as well as other authors, adopt the Ottawa Summit’s consensus definition: “Predatory journals and publishers are entities that prioritize self-interest at the expense of scholarship and are characterized by false or misleading information, deviation from best editorial/publication practices, lack of transparency, and/or use of aggressive and indiscriminate solicitation practices” [1].

Berger challenges the misconception that predatory publishing is primarily a problem of the Global South. Citing research by Moher and colleagues she notes that authors from the United States, including those affiliated with prestigious institutions, rank second in the world for publishing in suspected predatory journals [2]. The issue is global and affects a wide range of stakeholders: researchers, evaluators, funders, institutions, patients, and the public. It is particularly acute in STEM and biomedical fields where the pressures to publish rapidly are very high and consequences include wasted research funding, compromised scientific integrity, and misinformation. As Moher et al. argue, the erosion of trust in science and the loss of valuable data, often unarchived and unindexed, undermine the very rationale for human and animal research participation [2].

One of the most problematic aspects of predatory publishing is the lack of rigorous peer review. Yet, as Berger points out, peer review remains a “black box” even within mainstream publishing, complicating efforts to define and detect predatory practices.

Berger’s book is commendable for its clarity, depth, and commitment to equity. She provides a thorough historical account of predatory publishing, its entanglement with open access, and the disproportionate harm caused to authors and publishers in the Global South. Her analysis of Jeffrey Beall’s controversial list, criticized for its lack of transparency and for conflating low-resourced open access journals with predatory ones, is particularly thoughtful. Berger writes through an intentional lens of equity, diversity, and inclusion (EDI) and anti-racism, ensuring that marginalized voices and contexts are not unfairly maligned. This perspective makes the book an essential resource for understanding the issue in a nuanced and ethically grounded way.

Berger’s critique of neoliberalism and its influence on scholarly communication is a recurring theme. She argues that neoliberal values, efficiency, competition, and market-driven metrics, have infiltrated academia, reinforcing the conditions that allow predatory publishing to thrive.

This book is thoroughly researched and supported by extensive citations at the end of each chapter. It is organized into four parts:

## Part I: Background

This section includes three chapters that establish the foundation for the book’s arguments. Berger positions herself within the discourse and offers a history of open access and APCs, which are closely linked to the rise of predatory publishing. She provides a balanced overview of Jeffrey Beall’s contributions and controversies and examines how neoliberalism and flawed evaluation metrics contribute to the proliferation of low-quality publications. The section concludes with a discussion of various forms of publishing misconduct.

## Part II: Characteristics and research

Comprising three chapters, this section analyzes the motivations and behaviours of key actors. Chapter four focuses on journals, offering a detailed breakdown of predatory characteristics including a helpful summary table that serves as a practical reference. Chapter five explores the motivations of publishers and authors, presenting a nonjudgmental overview of why individuals, ranging from naïve early-career researchers to more strategic actors, submit to questionable journals. The personas outlined are particularly useful for understanding the spectrum of author behaviour. Chapter six turns to editors, spam solicitation tactics, and the role of funders as well as bibliometrics in perpetuating the cycle. Again, Berger provides a valuable summary of editor personas based on published research.

## Part III: An unbalanced landscape: the geopolitics of scholarly publishing

This section delves into the global inequalities that shape scholarly publishing. Berger critiques the dominant narrative, largely constructed by the Global North, that frames predatory publishing as a problem of the Global South. She presents evidence that the issue is international. The Bohannon Sting [3] operation is discussed in detail, revealing not only unethical publishing practices but also racial undertones in the execution of this study. Berger examines publishing practices in India, China, South Africa, and Sub-Saharan Africa, offering thoughtful insights into the challenges faced in these regions. She highlights the financial burden of APCs, noting that authors in the Global South often pay out-of-pocket at significantly higher rates than their Northern counterparts. This is based on flawed assumptions that the Global South primarily consumes rather than produces research. There is also discourse on publishing in the Global North and the successful infrastructure and community-based approach of Latin America.

## Part IV: Responses and solutions

The final section presents both structural and pedagogical solutions. Chapters 11 and 12 are particularly relevant to librarians, offering practical strategies for instruction and outreach. Berger discusses consultations, asynchronous learning tools, and workshops tailored to students and faculty. She generously shares links to handouts and instructional materials she has used, making this section a valuable resource for librarians involved in outreach and instruction.

While Jingfeng Xia’s *Predatory publishing* in 2022 covers similar ground [4], Berger’s book stands out for its anti-racist lens and its librarian-focused solutions. Berger’s work remains tightly focused on predatory publishing and does not venture into related issues such as paper mills [5] nor, unfortunately, the emerging role of AI in contributing to more research waste [6] or detecting scholarly misconduct [7].

## Concluding remarks

Overall, *Predatory publishing and global scholarly communications* is an important resource for understanding the nuanced and interconnected factors that make predatory publishing so pervasive and profitable. Berger’s writing is clear, well-researched, and grounded in ethical scholarship. The book consolidates a wealth of articles, resources, and tools including summaries from scholarly publishing societies and initiatives like Think. Check. Submit [8] into a single, accessible volume.

Berger identifies her ideal reader as someone “curious about scholarly communications and wants to deepen their knowledge of open access, scholarly assessment, peer review, and other topics such as the geopolitics of scholarship” [9]. This book delivers on that promise. It is highly recommended for professional development and is particularly useful for health sciences librarians working in academic, hospital, or research settings. With its practical insights, thoughtful analysis, and commitment to equity, this book represents excellent value and should be considered a core resource in any academic library.
